# Differences in topological properties of functional brain networks between menstrually-related and non-menstrual migraine without aura

**DOI:** 10.1007/s11682-020-00344-0

**Published:** 2020-07-23

**Authors:** Yutong Zhang, Tao Xu, Ziwen Wang, Dehua Li, Jiarong Du, Yi Wen, Yu Zhao, Huaqiang Liao, Fanrong Liang, Ling Zhao

**Affiliations:** 1 College of Acupuncture, Moxibustion and Tuina, Chengdu University of Traditional Chinese Medicine, 37 Shi’er Qiao Rd, Chengdu, 610075 Sichuan China; 2 Chengdu Integrated Traditional Chinese Medicine &Western Medicine Hospital, Chengdu, China; 3 Hospital of Chengdu University of Traditional Chinese Medicine, Chengdu, China

**Keywords:** Menstrually-related migraine without aura, Non-menstrual migraine without aura, Functional brain network, Topological properties

## Abstract

Menstrually-related migraine without aura refers to a specific type of migraine that is associated with the female ovarian cycle. Compared with non-menstrual migraine without aura, in menstrually-related migraine without aura, there are additional attacks of migraine outside of the menstrual period. Menstrually-related migraine without aura tends to be less responsive to acute treatment and more prone to relapse than non-menstrual migraine without aura. Currently menstrually-related migraine without aura is treated no differently from any other migraine but, the differences in the central mechanisms underlying menstrually-related migraine without aura and non-menstrual migraine without aura remain poorly understood. Here, using resting-state functional magnetic resonance imaging and graph theory approaches, we aimed to explore the differences in topological properties of functional networks in 51 menstrually-related migraine without aura patients and 47 non-menstrual migraine without aura patients. The major finding of our study was that significant differences in topological properties between the two groups were mainly evident in the nodal centrality of the inferior frontal gyrus and the thalamus. Nodal centrality in inferior frontal gyrus was negatively correlated with Headache Impact Test questionnaire scores in the menstrually-related migraine without aura patients. Partial least squares correlation analysis revealed enhanced correlations of inferior frontal gyrus to pain-related behavior in the non-menstrual migraine without aura group, while within the menstrually-related migraine without aura group these effects were non-significant. These results indicate that the regulatory mechanisms in the central nervous system may differ between the two subtypes of migraine. The results provide novel insights into the pathophysiology of different subtypes of migraine, and could help us to enhance their clinical diagnosis and treatment.

## Introduction

Migraine is an idiopathic headache disorder that is characterized as moderate to severe intensity, and is often associated with a combination of symptoms, including nausea, vomiting, tiredness, phonophobia, and photophobia (May 2009). Menstrually-related migraine without aura (MRM), a particular subtype of migraine without aura, includes additional migraines at menstruation, unlike non-menstrual migraine without aura (NMM) (Güven et al. 2017). Currently, the treatment for MRM is similar to that for NMM and mainly uses triptans and nonsteroidal anti-inflammatory drugs (NSAIDs) (Calhoun 2018), but MRM tends to be more resistant to treatment than NMM (Güven et al. 2017). However, the differences in pathophysiological mechanisms between MRM and NMM are not yet fully understood.

Sex hormones have long been linked with MRM as playing a possible mechanistic role in the condition, as they exert profound influences on the central nervous system (CNS) (Calhoun 2018) that may underlie MRM. A decline in estrogen levels in MRM patients may have a facilitating effect on the development of central sensitization, causing changes in the pain threshold of trigeminal neurons (Martin et al. 2007; Welch et al. 2006). These changes may be responsible for the stronger migraine attacks in patients with MRM rather than NMM (Güven et al. 2017). Additionally, the pathophysiology of MRM probably involves the activation of a number of different “pain processing networks” within the CNS, as well as vasodilatation of meningeal arteries secondary to calcitonin gene-related peptide release from the trigeminal nerve (Martin and Behbehani 2006). Ovarian hormones could modulate these pathways to increase the frequency, severity, or duration of MRM (Martin and Behbehani 2006). In light of such differences in MRM and NMM, an investigation of the pathogenic mechanisms in the two different subtypes of migraine may help the development of effective and innovative treatments for clinical application.

Neuroimaging approaches have been used to measure structural and functional brain changes in migraine patients (TJ & DW Schwedt and Dodick 2009), providing feasible, efficient, and noninvasive tools for investigating the pathophysiological mechanisms of migraine (Liu et al. 2015). Several brain regions in patients with migraine have shown structural or functional alterations, including prefrontal cortex (Gao et al. 2016), anterior cingulate cortex (Zhao et al. 2013), basal ganglia (BG) (Yuan et al. 2013), and insula(Liu et al. 2015). Further neuroimaging studies have pointed out that brain dysfunctions associated with migraine may not only occur in one or more isolated regions, but are also closely associated with distributed brain networks (Baliki et al. 2008; Liu et al. 2011). Topological alterations can reveal brain network dysfunction or reorganization in patients, which may affect information segregation and integration (Zhang et al. 2017). The investigation of the segregated and integrated processing in the female migraineurs’ brain network will significantly advance our understanding the progression of migraine and improve our ability to implement effective treatments.

The incidence and prevalence of migraine are significantly higher in women than men (Stewart et al. 2008), and functional magnetic resonance imaging (fMRI) results show that women have more vulnerable functional networks than men (May 2009). These sex-specific differences suggest that clinical treatment of women should differ to that of men (von Deneen et al. 2019). As a prevalent subtype of migraine in women, the symptoms of MRM are likely to be similar to those of NMM. However, the pathogenesis and clinical characteristics may partially differ between these two subtypes, which might contribute to variations in the underlying topological features of functional brain networks in each.

In the current study, we aimed to explore differences between MRM and NMM in terms of the topological properties of their underlying functional brain networks. To achieve this, resting-state fMRI and graph theory analysis (GTA) were used to investigate differences in the topological properties of brain networks between MRM and NMM patients.

## Methods

This study was approved by the Ethics Committee of the Hospital of Chengdu University of Traditional Chinese Medicine (TCM) and was conducted in accordance with the Declaration of Helsinki. All participants gave written, informed consent to participate after the experimental procedures had been fully explained and they were informed that they could stop participating at any time.

### Participants

51 MRM patients (age range, 18–50 years; mean, 33.04 ± 6.43 years) and 47 NMM patients (age range, 18–50 years; mean, 35.30 ± 9.43 years) were included in the final data analysis (Table 1). All patients were enrolled from the outpatient clinic of the Departments of Neurology and Gynecology in two clinical centers: (1) the Hospital of Chengdu University of TCM; and (2) Chengdu Integrated TCM &Western Medicine Hospital. Recruitment took place from June 2015 through August 2018. The diagnosis of MRM and NMM was established according to the ICHD III-beta criteria (Headache Classification Committee of the International Head] 2018). Participants who met all the following inclusion criteria were included in the study: (1) female, 18–50 years old, right-handed; (2) fulfilling criteria for migraine without aura; (3) migraine occurred on day 1 ± 2 of menstruation in at least two out of three menstrual cycles, and additionally at other times of the cycle for the MRM group; (4) migraine was experienced outside of the menstrual cycle in the NMM group; (5) history of migraine without aura for 6 months or more; and (6) regular menstrual cycle with a length of 28 ± 7 days. Patients with any of the following conditions were excluded: (1) macroscopic T2-visible brain lesions on magnetic resonance imaging (MRI) scans; (2) suffered from neurological diseases, immunodeficiency, bleeding disorders, or allergies; (3) MRI contraindications such as claustrophobia; (4) alcohol or drug abuse; (5) pregnancy, lactation, or plans to become pregnant within 6 months.

**Table 1:** Demographic characteristics of the participants

Characteristics	MRM (*n* = 51)	NMM (*n* = 47)	*p* value
Age (years)	33.04 ± 6.43	35.30 ± 9.43	0.173
Height (cm)	161.00 ± 7.54	158.96 ± 4.75	0.109
Weight (kg)	53.57 ± 6.30	54.46 ± 7.59	0.536
Disease duration (year)	7.13 ± 5.75	8.06 ± 5.74	0.426
Attack frequency (times)	5.29 ± 1.95	5.13 ± 2.83	0.730
VAS score	5.70 ± 2.06	5.27 ± 1.85	0.271
Duration of migraine attack (h)	13.07 ± 14.55	11.84 ± 11.51	0.646
HIT-6 score	64.82 ± 6.48	61.04 ± 8.69	0.016
MSQ score (restrictive subscale)	60.77 ± 13.07	61.88 ± 18.89	0.739
MSQ score (preventive subscale)	72.16 ± 17.04	73.26 ± 19.06	0.764
MSQ score (emotional functional subscale)	76.71 ± 15.24	74.47 ± 20.78	0.379

The data are shown as the mean ± SD. The independent-sample *t*-test was used to test the difference in each of the demographic characteristics between the two groups. Abbreviations: MRM, menstrually-related migraine without aura; NMM, non-menstrual migraine without aura; VAS, visual analogue scale; HIT-6, Headache Impact Test version 6. MSQ, Migraine-Specific Quality of Life Questionnaire

The drugs used for the prophylaxis of migraine were stopped 4 weeks before the experiment; however, participants were allowed to take emergency medicine when their pain was difficult to endure. Detailed information about the participants’ drug intake was recorded. All participants had been free from a typical migraine attack for at least 1 week prior to the MRI examination. MRI was performed during the periovulatory phase (days 12–16 of the menstrual cycle). Urine kits for luteinizing hormone were used to verify that the participants were in their periovulatory phase. A positive urine kit test result indicates a surge in the release of luteinizing hormone indicating impending ovulation. After scanning, all participants reported that they did not experience any headaches or migraines and remained awake during the measurement.

This study was approved by the supervision of the Sichuan Regional Ethics Review Committee on TCM (ethical approval number: 2015KL-004) and was registered in the Chinese Clinical Trial Registry (registration number: ChiCTR-IOR-15006648).

### Data acquisition

Information collected included age, height, and weight. All patients were required to keep a migraine diary to record visual analogue scale (VAS) scores (scale of 0–10, with 10 being the most intense pain imaginable), migraine attack frequency (number of times), and duration of migraine attack (h). The Headache Impact Test (HIT-6) questionnaire was adopted to assess the impact of migraine on the lives of the patients (Shin et al. 2008), and the Migraine-Specific Quality of Life Questionnaire (MSQ), a 14-item health-related quality of life questionnaire, was used to measure three dimensions of functional status specific to migraine (using preventive, restrictive, and emotional functional subscales)(Bagley et al. 2012).

MRI data were acquired with a GE Discovery MR750 3.0 T system with an eight-channel, phased-array head coil (General Electric, Milwaukee, WI, USA). The functional images were obtained with a single-shot gradient-echo echo-planar imaging (GRE-EPI) sequence with the following parameters: repetition time = 2000 ms; echo time = 25 ms; flip angle = 90°, field of view = 240 × 240 mm, data matrix = 64 × 64, slice thickness = 3 mm. During the whole functional scan, all participants were instructed to keep their eyes closed and stay awake during the entire session.

### Data preprocessing

The first 10 time points of functional data were discarded to eliminate non-equilibrium effects of magnetization and allow subjects to get used to the scanning environment. Data preprocessing of the remaining resting-state images was performed using the Data Processing & Analysis for Brain Imaging toolbox version 2.3 (DPABI v. 2.3; http://rfmri.org/dpabi). All data sets were processed using the following steps: 1) slice timing correction; 2) realignment to correct for spatial displacements due to head motion; 3) spatial normalization to the Montreal Neurological Institute echo-planar imaging template image and resampling to 3-mm isotropic voxels; 4) removal of the linear trend of the smoothed images; 5) regression of nuisance covariates, including 24 head movement parameters (Friston et al. 1996), cerebrospinal fluid signals, and white matter signals; 6) temporal band-pass filtering (0.01–0.08 Hz). Individuals with an estimated maximum displacement in any direction larger than 2 mm or head rotation larger than 2°were discarded from the study. Four patients in the NMM group were excluded from further analysis owing to excessive head movement (>2 mm) during fMRI scanning. No subjects exhibited head motions exceeding 2 mm of translation or 2°of rotation in any direction in the MRM group.

### Network construction

We constructed the functional network using the Graph Theoretical Network Analysis (GRETNA) toolbox (https://www.nitrc.org/projects/gretna/) (Wang et al. 2015); this method of network construction and calculation has been used in previous studies of brain networks (Huang et al. 2017).

For each subject, a functional connection matrix was constructed by defining nodes representing brain regions and edges representing interregional resting-state functional connectivity (RSFC) (Liu et al. 2015; Liu et al. 2012). To define the brain nodes of the functional networks, we used the Human Brainnetome Atlas, which divides the whole brain into 246 regions of interest (ROIs) (Fan et al. 2016). To measure the RSFC, the Pearson correlation coefficients between each pair of ROIs were calculated to form an *N* × *N* correlation matrix (where *N* is the number of ROIs, *N* = 246).

### Network metrics

GTA was employed to compute the topological properties of binarized functional connectivity networks at global and regional (nodal) levels. The global metrics examined in this study included the assortativity coefficient, hierarchical organization, global efficiency and small-world properties. The node metrics examined the nodal degree centrality (*DC*), which emphasizes the impact and significance of a network at the voxel level. It represents the most local and directly quantifiable centrality measure and reflects the properties of the functional brain network “hub” in relation to network information communication (Telesford et al. 2011).

We calculated the area under the curve (AUC) for each metric using the GRETNA toolbox(Wang et al. 2015). The AUC provides a summarized scalar for brain topological properties independent of single threshold selection and can sensitively detect topological alterations in neurological disorders (Huang et al. 2017; Lei et al. 2015). The matrix was thresholded at a value that yielded a binary undirected network of different link densities ranging from 0%–100% in 1% increments.

### Statistical analysis

Software (SPSS version 23.0; SPSS, Chicago, IL, USA) was used for demographic analysis. The independent-sample *t*-test was used to compare all demographic characteristics between the two groups. A significant difference was indicated when the *p* value was less than 0.05.

To determine whether there were significant differences in the topological properties of the functional brain networks between the MRM and NMM groups, two-sample *t* tests with a significance level of α = 0.05 were performed on the AUC of each network metric. To correct for multiple comparisons in each of these values, the Bonferroni correction was applied (*p* = 0.05/246, corresponding to a threshold of *p* = 0.0002).

To distinguish the potential effect of central mechanisms from that of clinical pathological parameters between the MRM and NMM groups, the demographic and clinical characteristics of the patients (including age, disease duration, attack frequency, VAS score, and duration of migraine attack) were used as covariates. After controlling for the demographic and clinical characteristics, we re-analyzed the AUC of each network metric. The Bonferroni correction was also used to correct for multiple comparisons (*p* = 0.05/246, corresponding to *p* = 0.0002).

### Partial least squares correlation analysis

To investigate the relationship between nodal degree centrality and pain-related behavioral variables (attack frequency, VAS score, HIT-6 score, and scores on all subscales of the MSQ), partial least squares correlation (PLSC) was used. PLSC is a correlational technique that analyzes associations between two sets of data (Krishnan et al. 2011), which can analyze the association between multiple behavioral measures and multimodal neuroimaging data in a single statistical model. The correlation matrix generated by PLSC is decomposed into latent variables using singular value decomposition to identify unique correlation patterns. PLSC is ideally suited to deriving underlying latent patterns in highly correlated dependent measures and may have higher sensitivity in identifying complex and potentially weak, yet interrelated patterns; thus, it has already been widely adapted and applied in neuroimaging studies (Keresztes et al. 2017).

Pain is a multi-dimensional subjective experience that involves the physical, emotional, and perceptual integration of noxious information (Blackburn-Munro and Blackburn-Munro 2003), and the PLSC has the advantage of being able to identify associations between pain-related behavioral data and brain activity. Overviews of the computational details of the method have been published by McIntosh and Laugh (2004), and Krishnan et al. (2011).

To determine the statistical significance of each latent variable in the PLSC, a permutation test was used (*p* < 0.001; 5000 permutation iterations). Five-thousand bootstrap samples were also created to assess the reliability of the effect at each node of the functional brain network. Brain and design saliences were recalculated for every bootstrap sample, yielding bootstrap ratios (BSRs; original saliences/bootstrap standard errors). We used a BSR threshold of ±3.3, which is equivalent to a *p* value of approximately 0.001.

## Results

### Demographic and clinical characteristics of subjects

The demographic characteristics of all subjects are summarized in Table 1. We found no statistical differences between the MRM and NMM groups in terms of their age, height, weight, disease duration, attack frequency, VAS score, or duration of migraine attack, or in any subscales of the MSQ. (all *p* > 0.05). Only HIT-6 showed a statistical difference between the two groups (*p* < 0.05; Table 1).

### Differences in network properties between groups

At the level of the global properties, no significant differences were observed in the AUCs of the hierarchical organization, global efficiency, small-world properties, or assortativity coefficient between the MRM and NMM groups (all *p* > 0.05; Table 2).

**Table 2:** Global network properties between the MRM and NMM groups

Global network properties	Corrected *p* value (MRM vs. NMM)
Assortativity coefficient	>0.05
Hierarchical organization	>0.05
Global efficiency	>0.05
Small-world properties	>0.05

The two-sample *t*-test was used to test the difference in global network properties between the two groups. Abbreviations: MRM, menstrually-related migraine without aura; NMM, non-menstrual migraine without aura

**Fig. 1:**
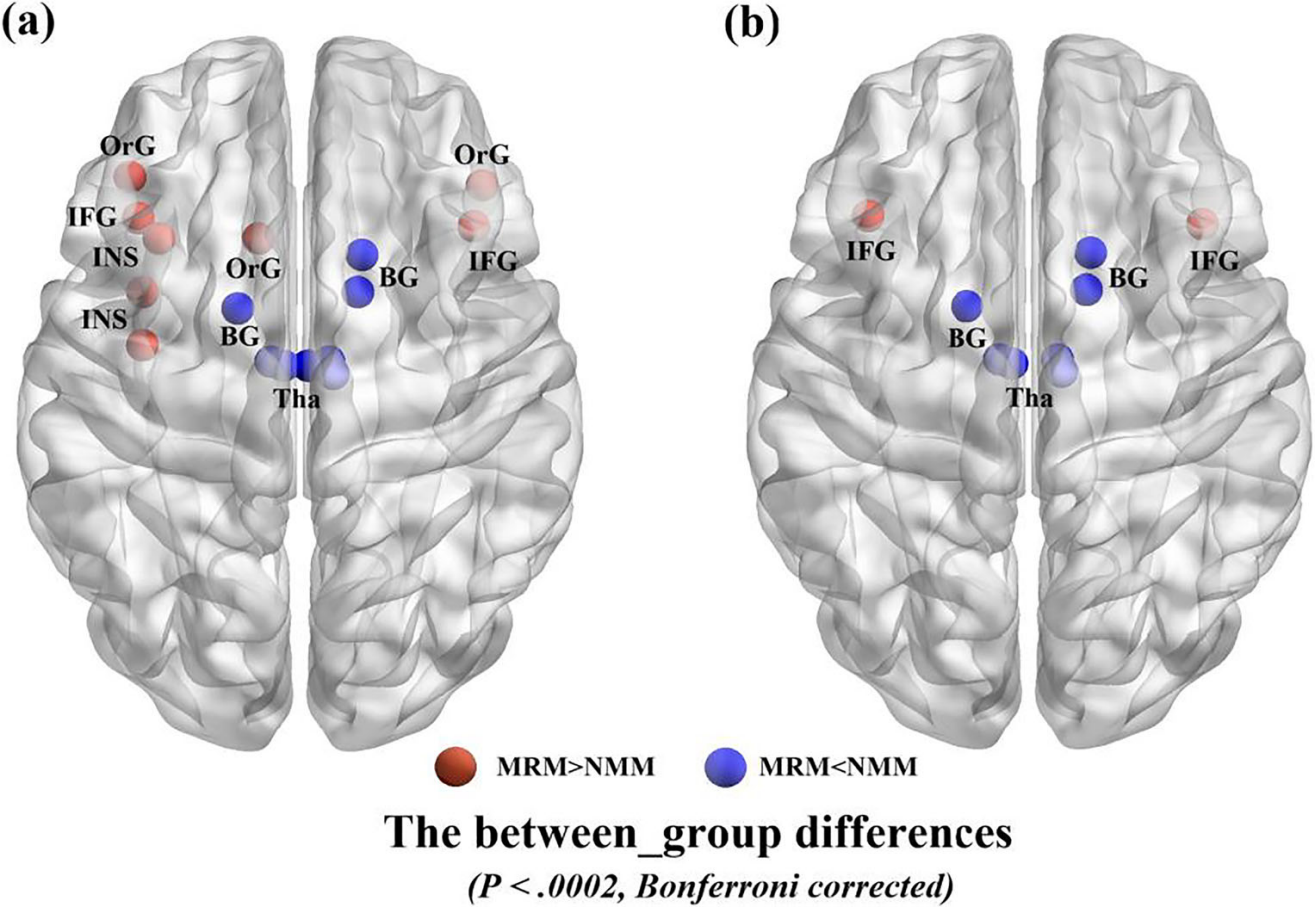
Significant between-group differences in the nodal degree centrality between the MRM and NMM groups. (**a**) Significant differences in the nodal degree centrality between the two groups before regression analysis. (**b**) Significant differences in the nodal degree centrality between the two groups after regression analysis. Regions colored in red have a significantly higher nodal degree for the MRM group, while regions colored in blue indicate a lower degree for the MRM group. Data shown were visualized with the BrainNet viewer (www.nitrc.org/projects/bnv). Abbreviations: BG, basal ganglia; IFG, inferior frontal gyrus; INS, insula; MRM, menstrually-related migraine without aura; NMM, non-menstrual migraine without aura; OrG, orbital gyrus; Tha, thalamus

**Fig. 2:**
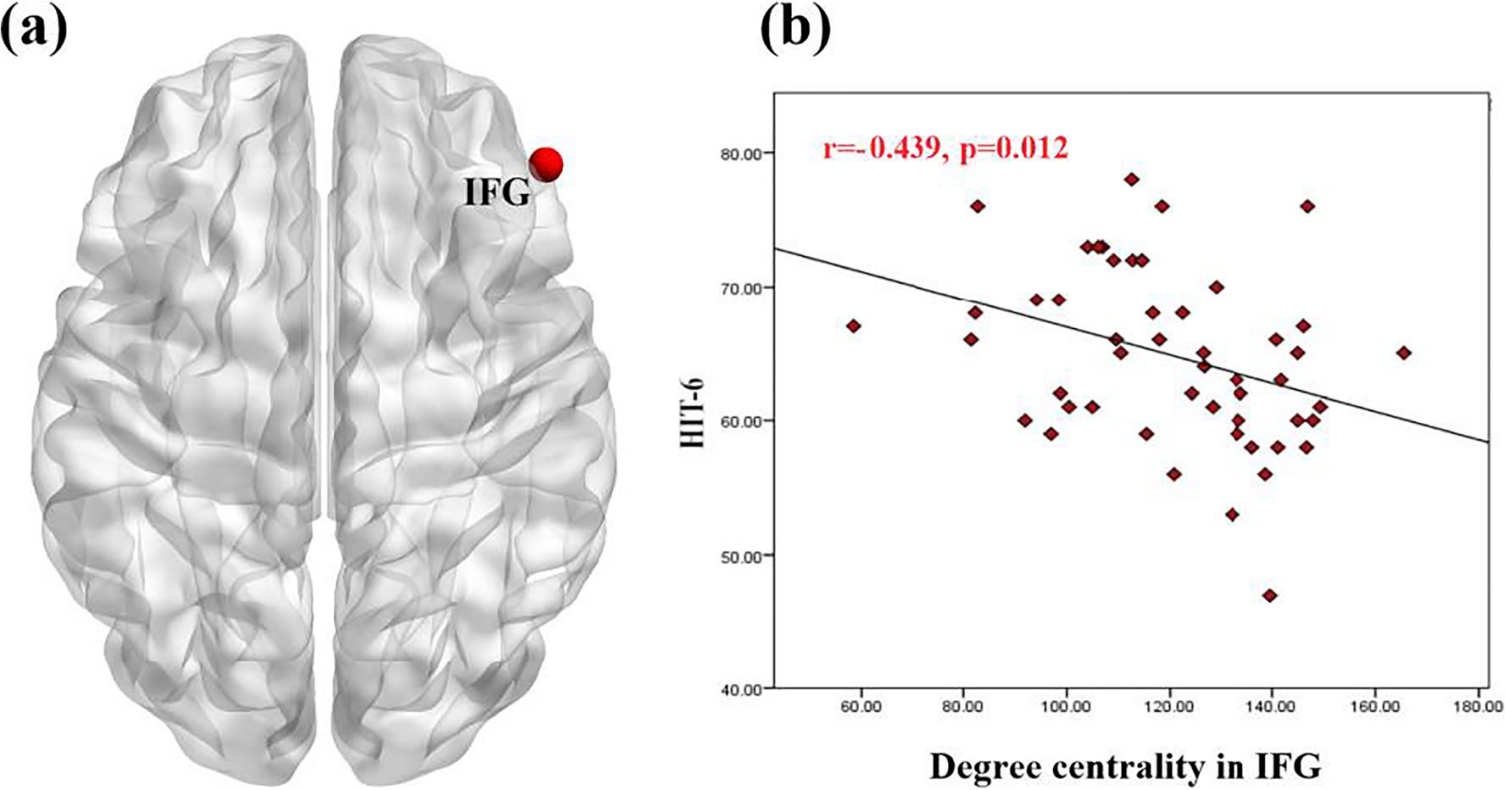
Correlation analysis between nodal degree in IFG and HIT-6 in the MRM group. (**a**) Brain region used for the correlation analysis shown. (**b**) Degree centrality in the IFG was negatively correlated with HIT-6 in the MRM patients (r = −0.439, *p* = 0.012). Abbreviations: HIT-6, the headache impact test; IFG, inferior frontal gyrus; MRM, menstrually-related migraine without aura; NMM, non-menstrual migraine without aura

Regarding the nodal *DC*, compared with the NMM group, the MRM group had higher a degree mainly in the insula, inferior frontal gyrus (IFG), and orbital gyrus (OrG), and a lower degree in the thalamus and BG. After controlling for the demographic and clinical characteristics, group differences between MRM and NMM patients were no longer present in the insula, but they persisted within the IFG, thalamus, and BG (Fig. 1; Table 3). Additionally, we further investigated the association between the above regions and HIT-6 scores using Pearson correlation analysis. We found that the *DC* in the IFG of MRM patients was negatively correlated with their HIT-6 scores (*p* = 0.012, r = −0.349; Fig. 2), but an equivalent correlation was not found in the NMM group.

**Table 3:** Significant changes in the degree centrality between the MRM and NMM groups

Regions	Degree centrality			
	MRM	NMM	Corrected *p*-value^a^ (MRM vs. NMM)	Corrected *p*-value^b^ (MRM vs. NMM)
	Mean ± SD	Mean ± SD		
INS_l	126.0 ± 17.6	104.7 ± 21.9	<0.0002	–
Tha_l	91.7 ± 38.1	129.6 ± 29.4	<0.0002	<0.0002
Tha_r	82.6 ± 34.7	116.4 ± 37.0	<0.0002	<0.0002
OrG_l	131.7 ± 18.8	114.3 ± 18.3	<0.0002	<0.0002
OrG_r	134.4 ± 18.9	117.9 ± 18.8	<0.0002	<0.0002
IFG_l	128.9 ± 20.0	108.9 ± 25.3	<0.0002	<0.0002
IFG_r	128.4 ± 17.0	109.0 ± 23.4	<0.0002	<0.0002
BG_l	92.4 ± 28.4	127.2 ± 25.9	<0.0002	<0.0002
BG_r	91.7 ± 27.7	123.5 ± 29.0	<0.0002	<0.0002

The data are shown as the mean ± SD. ^a^ before regression analysis; ^b^ after regression analysis. The two-sample *t*-test was used to test the difference in degree centrality between the two groups. The Bonferroni correction was used to correct for multiple comparisons. Abbreviations: MRM, menstrually-related migraine without aura; NMM, non-menstrual migraine without aura

Taken together, after controlling for the demographic and clinical characteristics, these results indicate the group differences in nodal degree between the MRM and NMM patients were no longer present in the insula, while those in the IFG, thalamus, and BG persisted. Nodal centrality in the IFG of MRM patients was negatively correlated with their HIT-6 scores (*p* < 0.05).

### Results of PLSC analysis

**Fig. 3:**
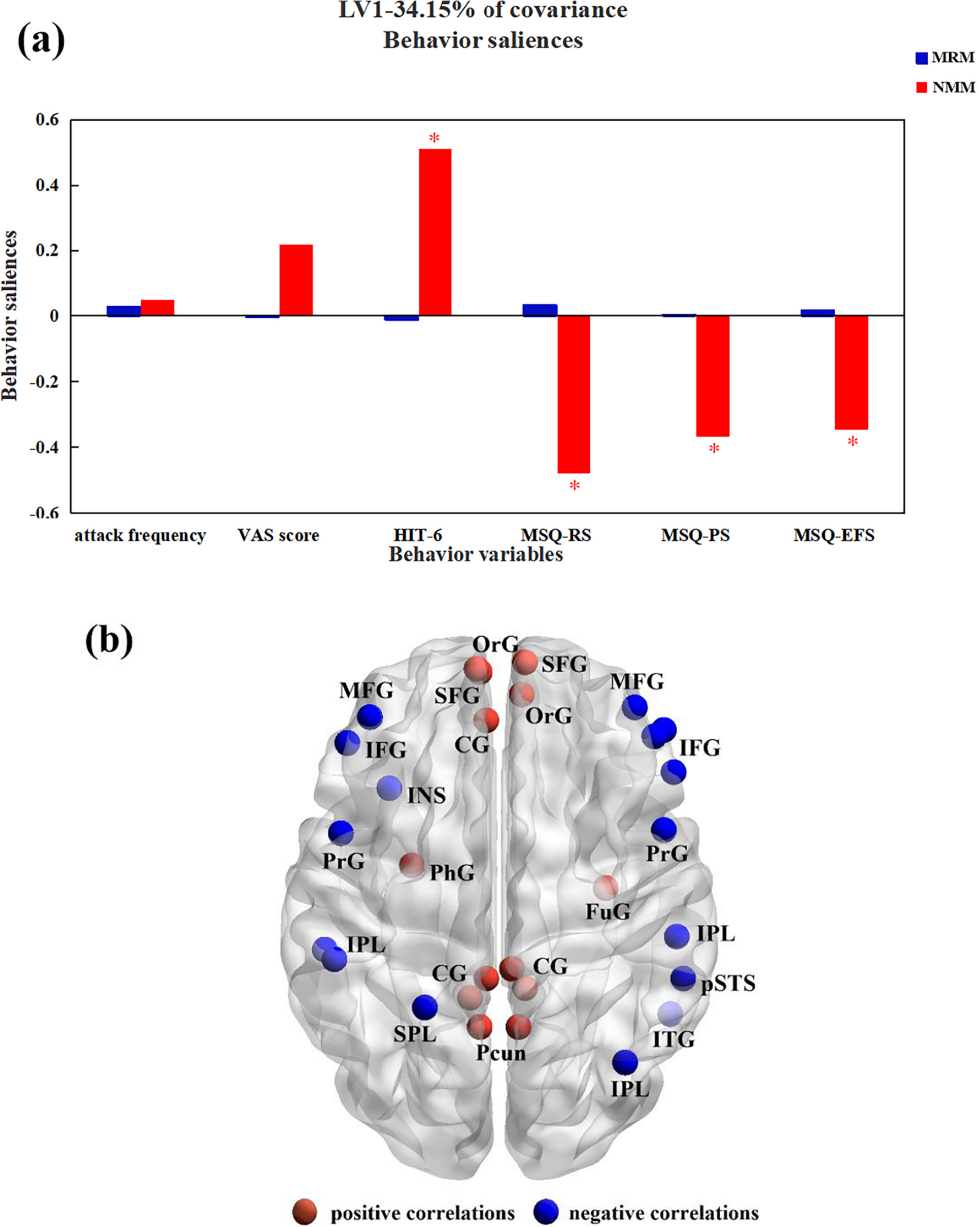
PLSC results for the association of behavioral variables and degree centrality in the MRM and NMM groups. (**a**) Associations between degree centrality and behavioral variables in the MRM and NMM groups. *5000-times permutation test. Abbreviations: LV, latent variable; MRM, menstrually-related migraine without aura; MSQ, Migraine-Specific Quality of Life Questionnaire; MSQ-EFS: MSQ score (emotional functional subscale); MSQ-PS, MSQ score (preventive subscale); MSQ-RS, MSQ score (restrictive subscale); NMM, non-menstrual migraine without aura. (**b**) Brain regions with significant associations with behavioral variables. Colored regions show the brain regions significantly positively correlated (red) or significantly negatively correlated (blue) with behavioral variables between the two groups. Data shown were visualized with the BrainNet viewer. Abbreviations: CG, cingulate gyrus; FuG, fusiform gyrus; IFG, inferior frontal gyrus; IPL, inferior parietal lobule; ITG, inferior temporal gyrus; INS, insula; PhG, parahippocampal gyrus; MFG, middle frontal gyrus; OrG, orbital gyrus; Pcun, precuneus; PrG, precentral gyrus; pSTS, posterior superior temporal sulcus; SFG, superior frontal gyrus; SPL, superior parietal lobule

In terms of the relationship between nodal degree and pain-related behavior, only one latent variable showed a significant contribution to the covariance according to a 5000-fold permutation test (34.15%, *p* < 0.001). The behavioral saliences of the significant latent variable (LV) are shown in Fig. 3a. According to our findings, scores from the HIT-6 and from all subscales of the MSQ significantly contributed to the brain-behavior correlation in the NMM group, while equivalent results were not found in the MRM group.

All significant brain regions showing an association with pain-related behavior are shown in Fig. 3b. For the NMM group, positive associations between HIT-6 score and nodal degree were found in the OrG, superior frontal gyrus, fusiform gyrus, parahippocampal gyrus, precuneus, cingulate gyrus. Negative correlations were found in the IFG, middle frontal gyrus, precentral gyrus, inferior temporal gyrus, posterior superior temporal sulcus, superior parietal lobule, inferior parietal lobule, and insula. The above association was opposite for all subscales of the MSQ.

## Discussion

In this study, we investigated differences in the topological properties of functional brain networks between MRM and NMM patients, and further explored differences in nodal centrality between the two groups. The major finding of our study is that the topological properties of the functional network show significant differences between the two groups, mainly in terms of the nodal centrality. Combined with regression analysis, our results suggest that the IFG, thalamus, and BG play important roles in the specific pathophysiology of MRM.

The IFG exhibited a higher nodal centrality in MRM patients than NMM patients. Nodal centrality is a powerful measure for assessing the relative importance of a node in a network, and has been applied to evaluating the information integration ability of an individual brain region within cortical networks (Liu et al. 2012). Our results indicate that there may be a difference between the two groups in terms of information transmission of the IFG in the functional brain network at rest. Furthermore, previous studies of pain found that the IFG not only plays a role in the cognitive modulation of pain (Bingel and Tracey 2008), but is also involved in memory retrieval (Denkova et al. 2013) and emotional regulation of pain (Ochsner et al. 2012). In our results, nodal centrality in the IFG was negatively correlated with HIT-6 score only in the MRM patients. The HIT-6 was used to assess the impact of headache on the quality of life of the respondents (Martynowicz et al. 2019). The questions within this test cover the content areas found in widely used measures of headache impact, including pain, social-role limitations, cognitive functioning, psychological distress, and vitality (Kosinski et al. 2003). These findings further indicate that the function of the IFG may relate to regulation of pain and emotion processing, and that the IFG would be a crucial component of the central mechanisms of MRM. However, no significant correlation was found for NMM, suggesting this condition may be mediated by other networks.

In the present study, we found the thalamus exhibited lower nodal centrality in MRM patients compared with NMM patients. Circulating sex hormones may modulate pain sensitivity by affecting neural activity (Veldhuijzen et al. 2013); when estrogen levels are declining rapidly or at very low levels, there is the greatest intensity of pain in premenstrual women with migraine (Maurer et al. 2016). The thalamus is a nuclear complex located in the diencephalon, the deep, central brain structure located between the midbrain and cortex, which plays a key role in the transmission of nociceptive information to the cerebral cortex (Yen and Lu 2013). Neuroimaging studies have reported that patients with migraine show white matter lesions (Yu et al. 2013), gray matter atrophy (Zhao et al. 2014), and abnormal spontaneous functional activity (Xue et al. 2013) in thalamus. Some studies have also found that thalamus is activated during the pain stimulation period of the luteal phase, during which the pain threshold is lower for most pain stimulation procedures (Choi et al. 2006). Current evidence suggests that the relationship of the neural mechanisms of MRM to sex hormone levels is likely to be diverse and complex. As a significant between-group difference was found in the functional integration of the thalamus, our findings indicate that the MRM and NMM groups may differ in terms of the thalamus-related pain processing circuit. This difference in the thalamus between the two groups may relate to the fact that migraine attacks occur at different phases in the menstrual cycle in MRM compared to NMM. A prior study reported that hormonal factors may have a facilitating effect on the development of central sensitization (Güven et al. 2017), which enhances central sensitization of the trigeminal system after a decline in estrogen levels in MRM patients (Martin et al. 2007). The activation of the trigeminal system has been found to be associated with ascending nociceptive transmission via the trigemino-thalamo-cortical pathway (Sokolov et al. 2014). Therefore, we speculate that estrogen levels may affect functional brain networks in MRM patients through the central sensitization of the thalamus.

Previous work suggests that the BG is involved in most aspects of pain processing, including sensory-discriminative, emotional/affective, and cognitive dimensions of pain, and in pain modulation (Borsook et al. 2010). With regard to migraine, brain imaging studies have shown that the BG serves as a sensitive neuroimaging marker, reflecting the disease duration of female migraine patients without aura(Gao et al. 2016) and that BG activity in response to pain is altered by an increasing frequency of migraine (Maniyar and Goadsby 2013). Migraine attacks in MRM not only occur at menstruation, but also additional attacks can occur at other times during the cycle. The altered nodal centrality in BG may further indicate why migraine attacks are more frequent in MRM than in NMM. Moreover, the alterations in nodal centrality may affect pain processing or modulation pathways, and may even affect functional integration or segregation processing within the functional network (Zhang et al. 2017). In our study, the functional network of the MRM group had a lower nodal degree in the BG than the NMM group, suggesting that pain processing or modulation pathways may be disrupted in MRM, which in line with previous research that the BG might be implicated in impaired pain processing and modulatory processes in migraineurs without aura (Yuan et al. 2013).

With PLSC analysis, it is possible to explore the relationship between multiple predictor variables and multiple response variables, enabling complex relationships within the data to be evaluated (Weaving et al. 2019). In our study, we used PLSC to analyze the relationship between nodal degree centrality and pain-related behavioral variables between the two groups. Our findings indicate the regulatory mechanisms in the CNS may differ between the two subtypes of migraine. The different central mechanisms occurring in NMM may correlate with pain-related behavioral variables, while those in MRM might be affected by various determinants, including sex hormone fluctuations and genetic variance. Thus, further investigations are needed to elucidate the underlying neural mechanisms of MRM.

There are several limitations to this study. First, because hormonal testing was not carried out, we were unable to compare hormone results between patients in the two groups. In view of the important role of hormones in MRM, hormone testing should be introduced in future studies to explore the influence of hormones on the neuroimaging results. Second, currently there are numerous fMRI studies that have investigated differences between migraine patients and healthy controls. To explore the different central mechanisms underlying the two subtypes of migraine, we chose not to include a group of healthy subjects as a control, which may have prevented us from examining the difference between the two subtypes in depth. Third, the MRM group showed a strong trend compared with the NMM group (Table 1), but because of the limited sample size, no statistical difference was found between the two groups. Further longitudinal fMRI studies will be required to establish and confirm the findings of the current study.

## Conclusions

Our findings reveal the different topological properties of functional brain networks in MRM and NMM. Group differences in nodal degree between the two subtypes of migraine were found in the IFG, thalamus, and BG. Combined with regression and PLSC analysis, the results indicate that the regulatory mechanisms in the CNS may differ between the two subtypes of migraine. MRM might be affected by the IFG and thalamus, while the pathophysiology of NMM may be associated with pain-related behavioral variables. When considering treatment options in this patient population, it is important to distinguish NMM from MRM, as the latter includes additional migraines at other times of the month, and it can influence the choice of treatment.

## References

[CR1] Bagley CL, Rendas-Baum R, Maglinte GA, Yang M, Varon SF, Lee J, Kosinski M (2012). Validating migraine-specific quality of life questionnaire v2.1 in episodic and chronic migraine. Headache.

[CR2] Baliki MN, Geha PY, Apkarian AV, Chialvo DR (2008). Beyond feeling: Chronic pain hurts the brain, disrupting the default-mode network dynamics. The Journal of Neuroscience.

[CR3] Bingel U, Tracey I (2008). Imaging CNS modulation of pain in humans. Physiology.

[CR4] Blackburn-Munro G, Blackburn-Munro R (2003). Pain in the brain: Are hormones to blame?. Trends in Endocrinology and Metabolism: TEM.

[CR5] Borsook D, Upadhyay J, Chudler EH, Becerra L (2010). A key role of the basal ganglia in pain and analgesia--insights gained through human functional imaging. Molecular Pain.

[CR6] Calhoun AH (2018). Understanding Menstrual Migraine. Headache.

[CR7] Choi JC, Park SK, Kim YH, Shin YW, Kwon JS, Kim JS, Kim JW, Kim SY, Lee SG, Lee MS (2006). Different brain activation patterns to pain and pain-related unpleasantness during the menstrual cycle. Anesthesiology.

[CR8] von Deneen KM, Zhao L, Liu J (2019). Individual differences of maladaptive brain changes in migraine and their relationship with differential effectiveness of treatments. Brain Science Advances.

[CR9] Denkova E, Dolcos S, Dolcos F (2013). The effect of retrieval focus and emotional valence on the inferior frontal cortex activity during autobiographical recollection. Frontiers in Behavioral Neuroscience.

[CR10] Fan L, Li H, Zhuo J, Zhang Y, Wang J, Chen L, Yang Z, Chu C, Xie S, Laird AR, Fox PT, Eickhoff SB, Yu C, Jiang T (2016). The human Brainnetome atlas: A new brain atlas based on connectional architecture. Cerebral Cortex.

[CR11] Friston KJ, Williams S, Howard R, Frackowiak RS, Turner R (1996). Movement-related effects in fMRI time-series. Magnetic Resonance in Medicine.

[CR12] Gao Q, Xu F, Jiang C, Chen Z, Chen H, Liao H, Zhao L (2016). Decreased functional connectivity density in pain-related brain regions of female migraine patients without aura. Brain Research.

[CR13] Güven B, Güven H, Çomoğlu S (2017). Clinical characteristics of menstrually related and non-menstrual migraine. Acta Neurologica Belgica.

[CR14] Headache Classification Committee of the International Headache Society (IHS) (2018). The International Classification of Headache Disorders, 3rd edition. Cephalalgia.

[CR15] Huang L, Dong HJ, Wang X, Wang Y, Xiao Z (2017). Duration and frequency of migraines affect cognitive function: Evidence from neuropsychological tests and event-related potentials. The Journal of Headache and Pain.

[CR16] Keresztes A, Bender AR, Bodammer NC, Lindenberger U, Shing YL, Werkle-Bergner M (2017). Hippocampal maturity promotes memory distinctiveness in childhood and adolescence. Proceedings of the National Academy of Sciences of the United States of America.

[CR17] Kosinski M, Bayliss MS, Bjorner JB, Ware JE, Garber WH, Batenhorst A (2003). A six-item short-form survey for measuring headache impact: The HIT-6. Quality of Life Research.

[CR18] Krishnan A, Williams LJ, McIntosh AR, Abdi H (2011). Partial least squares (PLS) methods for neuroimaging: A tutorial and review. NeuroImage.

[CR19] Lei D, Li K, Li L, Chen F, Huang X, Lui S, Li J, Bi F, Gong Q (2015). Disrupted functional brain Connectome in patients with posttraumatic stress disorder. Radiology.

[CR20] Liu J, Qin W, Nan J, Li J, Yuan K, Zhao L, Zeng F, Sun J, Yu D, Dong M, Liu P, von Deneen KM, Gong Q, Liang F, Tian J (2011). Gender-related differences in the dysfunctional resting networks of migraine suffers. PLoS One.

[CR21] Liu J, Zhao L, Lei F, Zhang Y, Yuan K, Gong Q, Liang F, Tian J (2015). Disrupted resting-state functional connectivity and its changing trend in migraine suffers. Human Brain Mapping.

[CR22] Liu J, Zhao L, Li G, Xiong S, Nan J, Li J, Yuan K, von Deneen KM, Liang F, Qin W, Tian J (2012). Hierarchical alteration of brain structural and functional networks in female migraine sufferers. PLoS One.

[CR23] Maniyar FH, Goadsby PJ (2013). Functional imaging in chronic migraine. Current Pain and Headache Reports.

[CR24] Martin VT, Behbehani M (2006). Ovarian hormones and migraine headache: Understanding mechanisms and pathogenesis--part I. Headache.

[CR25] Martin VT, Lee J, Behbehani MM (2007). Sensitization of the trigeminal sensory system during different stages of the rat estrous cycle: Implications for menstrual migraine. Headache.

[CR26] Martynowicz H, Smardz J, Michalek-Zrabkowska M, Gac P, Poreba R, Wojakowska A, Mazur G, Wieckiewicz M (2019). Evaluation of relationship between sleep bruxism and headache impact Test-6 (HIT-6) scores: A Polysomnographic study. Frontiers in Neurology.

[CR27] Maurer AJ, Lissounov A, Knezevic I, Candido KD, Knezevic NN (2016). Pain and sex hormones: A review of current understanding. Pain Management.

[CR28] May A (2009). New insights into headache: An update on functional and structural imaging findings. Nature Reviews Neurology.

[CR29] McIntosh Anthony Randal, Lobaugh Nancy J. (2004). Partial least squares analysis of neuroimaging data: applications and advances. NeuroImage.

[CR30] Ochsner, K. N., Silvers, J. A., & Buhle, J. T. (2012). Functional imaging studies of emotion regulation: A synthetic review and evolving model of the cognitive control of emotion. *Annals of the New York Academy of Sciences, 1251*, E1–E24. 10.1111/j.1749-6632.2012.06751.x PMC4133790 23025352

[CR31] Shin HE, Park JW, Kim YI, Lee KS (2008). Headache impact Test-6 (HIT-6) scores for migraine patients: Their relation to disability as measured from a headache diary. Journal of Clinical Neurology.

[CR32] Sokolov AY, Lyubashina OA, Sivachenko IB, Panteleev SS (2014). Effects of intravenous metamizole on ongoing and evoked activity of dura-sensitive thalamic neurons in rats. European Journal of Pharmacology.

[CR33] Stewart WF, Wood C, Reed ML, Roy J, Lipton RB (2008). Cumulative lifetime migraine incidence in women and men. Cephalalgia.

[CR34] Telesford QK, Simpson SL, Burdette JH, Hayasaka S, Laurienti PJ (2011). The brain as a complex system: Using network science as a tool for understanding the brain. Brain Connectivity.

[CR35] Schwedt TJ, Dodick DW (2009). Advanced neuroimaging of migraine. Lancet Neurology.

[CR36] Veldhuijzen DS, Keaser ML, Traub DS, Zhuo J, Gullapalli RP, Greenspan JD (2013). The role of circulating sex hormones in menstrual cycle-dependent modulation of pain-related brain activation. Pain.

[CR37] Wang J, Wang X, Xia M, Liao X, Evans A, He Y (2015). GRETNA: A graph theoretical network analysis toolbox for imaging connectomics. Frontiers in Human Neuroscience.

[CR38] Weaving D, Jones B, Ireton M, Whitehead S, Till K, Beggs CB (2019). Overcoming the problem of multicollinearity in sports performance data: A novel application of partial least squares correlation analysis. PLoS One.

[CR39] Welch KM, Brandes JL, Berman NE (2006). Mismatch in how oestrogen modulates molecular and neuronal function may explain menstrual migraine. Neurological Sciences.

[CR40] Xue, T., Yuan, K.,. P., Cheng, L., Zhao, L., Zhao, D., Yu, et al. (2013). Alterations of regional spontaneous neuronal activity and corresponding brain circuit changes during resting state in migraine without aura. *NMR in Biomedicine, 26*(9), 1051–1058. 10.1002/nbm.2917 23348909

[CR41] Yen CT, Lu PL (2013). Thalamus and pain. Acta Anaesthesiologica Taiwanica : Official Journal of the Taiwan Society of Anesthesiologists.

[CR42] Yu D, Yuan K, Zhao L, Dong M, Liu P, Yang X, Liu J, Sun J, Zhou G, Xue T, Zhao L, Cheng P, Dong T, von Deneen KM, Qin W, Tian J (2013). White matter integrity affected by depressive symptoms in migraine without aura: A tract-based spatial statistics study. NMR in Biomedicine.

[CR43] Yuan K, Zhao L, Cheng P, Yu D, Zhao L, Dong T, Zhao L, Cheng P, Yu D, Zhao L, Dong T, Xing L, Bi Y, Yang X, von Deneen KM, Liang F, Gong Q, Qin W, Tian J (2013). Altered structure and resting-state functional connectivity of the basal ganglia in migraine patients without aura. The Journal of Pain.

[CR44] Zhang J, Su J, Wang M, Zhao Y, Zhang QT, Yao Q, Lu H, Zhang H, Li GF, Wu YL, Liu YS, Liu FD, Zhuang MT, Shi YH, Hou TY, Zhao R, Qiao Y, Li J, Liu JR, du X (2017). The posterior insula shows disrupted brain functional connectivity in female Migraineurs without Aura based on Brainnetome atlas. Scientific Reports.

[CR45] Zhao L, Liu J, Dong X, Peng Y, Yuan K, Wu F, Sun J, Gong Q, Qin W, Liang F (2013). Alterations in regional homogeneity assessed by fMRI in patients with migraine without aura stratified by disease duration. The Journal of Headache and Pain.

[CR46] Zhao L, Liu J, Yan X, Dun W, Yang J, Huang L, Kai Y, Yu D, Qin W, Jie T, Liang F (2014). Abnormal brain activity changes in patients with migraine: A short-term longitudinal study. Journal of Clinical Neurology.

